# Web-Assisted Tobacco Interventions: Empowering Change in the Global Fight for the Public’s (e)Health

**DOI:** 10.2196/jmir.1171

**Published:** 2008-11-25

**Authors:** Cameron D Norman, Scott McIntosh, Peter Selby, Gunther Eysenbach

**Affiliations:** ^7^Department of Health PolicyManagement and EvaluationUniversity of TorontoTorontoCanada; ^6^Centre for Global eHealth InnovationUniversity Health NetworkTorontoCanada; ^5^Department of PsychiatryUniversity of TorontoTorontoCanada; ^4^Department of Family and Community MedicineUniversity of TorontoTorontoCanada; ^3^Centre for Addiction and Mental HealthTorontoCanada; ^2^Department of Community and Preventive Medicine University of RochesterRochester, NYUSA; ^1^Dalla Lana School of Public HealthUniversity of TorontoTorontoCanada

**Keywords:** Web-assisted tobacco intervention, public health, eHealth, tobacco control, tobacco cessation, prevention, population health, Internet

## Abstract

Tobacco control in the 21^st^ century faces many of the same challenges as in the past, but in different contexts, settings and enabled by powerful new tools including those delivered by information and communication technologies via computer, videocasts, and mobile handsets to the world. Building on the power of electronic networks, Web-assisted tobacco interventions (WATI) provide a vehicle for delivering tobacco prevention, cessation, social support and training opportunities on-demand and direct to practitioners and the public alike. The Framework Convention on Tobacco Control, the world’s first global public health treaty, requires that all nations develop comprehensive tobacco control strategies that include provision of health promotion information, population interventions, and decision-support services. WATI research and development has evolved to provide examples of how eHealth can address all of these needs and provide exemplars for other areas of public health to follow. This paper discusses the role of WATI in supporting tobacco control and introduces a special issue of the Journal of Medical Internet Research that broadens the evidence base and provides illustrations of how new technologies can support health promotion and population health overall, empowering change and ushering in a new era of public eHealth.

## Welcome to the Theme Issue on Web-Assisted Tobacco Interventions

Tobacco control is at a crossroads. On the positive side, tobacco-related diseases are being prevented and treated better than ever. For example, in the United States, the overall cancer death rate decreased by 12% between 1991 and 2003. A significant proportion of this decline (40%) is not due to breakthroughs in molecular medicine, gene therapy, or other highly technical treatments, but to a behavioral intervention: smoking cessation [1]. This effect is evident in all cancers put together, not just lung cancer.

 While positive, the gains shown in countries where tobacco use was first widespread (and where tobacco control efforts first took hold) are made less visible by the threat that tobacco continues to pose globally. Tobacco was responsible for more than 100 million deaths worldwide in the 20^th^ century and is forecast to kill at least one billion more in the century to come. More troubling perhaps is that 80% of such deaths are projected to occur in the developing world [2], where tobacco companies have focused their marketing efforts [3]. Half of the current smokers today (about 650 million people) will die as a result of tobacco use, with tobacco use accounting for the premature death of 4.9 million people worldwide [4].

To profile the state of tobacco and its current threat and impact on population health, the World Health Organization identified six strategies that are essential to reducing the burden of tobacco worldwide: 1) *M*onitor tobacco use and prevention policies, 2) *P*rotect people from tobacco smoke, 3) *O*ffer help to quit tobacco use, 4) *W*arn about the dangers of tobacco, 5) *E*nforce bans on tobacco advertising, promotion and sponsorship, and 6) *R*aise taxes on tobacco (MPOWER). Combined, these MPOWER strategies comprise the comprehensive tobacco control strategy that is reflected in the Framework Convention on Tobacco Control [5], the world’s first global public health treaty. What the report does not articulate – indeed what tobacco control struggles with as a whole – is how strategies like these can be carried out in practice and identifying the methods that are effective, transnational in scope, efficient in their use of scarce resources, and accessible to those that need them.

It is here that technology-enabled information tools hold promise. Web-assisted tobacco interventions (WATI) represent the vanguard of a new method of engaging the public, health professionals, and researchers alike in tobacco control as part of a greater *public eHealth* strategy. Technologies such as interactive websites, wireless phones, and handheld computers have shown promise as tools to support smoking prevention and cessation [6-13], health policy development [14] and knowledge translation for health promotion [15]. To support establishment of this nascent transdisciplinary field of research and practice, the WATI Initiative was developed in 2004 to support the development, study, and implementation of technology-delivered interventions to support tobacco control [16]. The *Web* part of WATI refers not only to interventions that are accessible from a desktop and the World Wide Web, but also to other networked technologies such as wireless phones or hybrid mobile devices such as the *iPhone*, *Blackberry* or other ‘smart phone’ handsets. As information becomes more tightly integrated across technologies this will remain an important distinction, particularly given the blurring of technologies that allow tools to be accessed across platforms and devices.

WATI resources have focused on four key areas: 1) cessation, 2) prevention, 3) social support, and 4) professional development and training. They can be used as a stand-alone intervention, a complement to other (mostly non-Internet) resources, or as an integrated component within a larger intervention [17]. WATI-focused research has received considerable attention within the eHealth field, including many publications in this journal over the past 10 years [8, 18-26]. WATI resources are becoming popular in part because the high proximal (and rising) levels of Internet access in many countries [27] and the prospect that, with wide reaching accessibility, a small shift in behavior attributed to a Web intervention can readily translate into a large population health effect.

Never before has this been more important considering the prognosis outlined by the WHO. Yet in spite of such promise, eHealth’s principal challenge is ensuring the distribution of benefits are equitable and do not simply confer advantages to those who already have resources [28]. It also means considering how eHealth tools used in a developed nation may not be the same ones we employ in the developing world to address tobacco control. For example, as anyone who has travelled widely can see, wireless phones are used for much more than talking (such as banking or ecommerce) in Africa, Asia and Europe, but much less so in North America. Likewise, penetration of smart phone technology through tools such as the Blackberry and others remain largely confined to the US, Canada and Western Europe. But in both cases, the use of technology is rapidly transforming the way people interact locally and globally.

**Figure 1:**
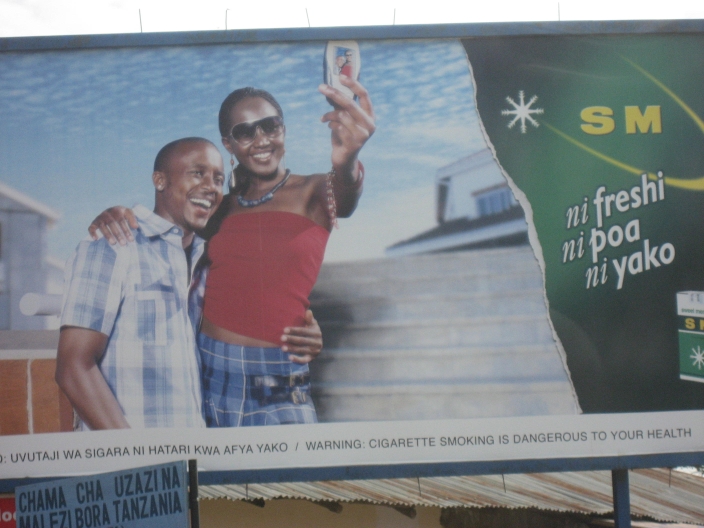
Cigarette billboard advertisement, Arusha, Tanzania, January 2007

However, these changes are providing avenues for tobacco promotion as well as control. Advertisements, such as the one depicted in Figure 1 from one of the author’s recent visits to Tanzania, illustrate ways in which technology is being blended into promotions for tobacco products. Here, a phone camera is used as a technology-friendly way to accent cigarette promotions. In other areas, the Internet has provided a transnational avenue towards the establishment of ‘dark markets’ where the tobacco industry has sought to exploit, reaching populations that are illegal to sell to, such as youth, in places where they are otherwise legally forbidden to advertise [29-31] and where the regulations governing Internet communications are often unclear.

The rise of new tools that blend photography, video, text, and voice and move information from stationary computers to mobile technologies have enabled countries that had no access to remote resources to leapfrog forward in the telecommunications evolution [32]. Where the digital divide was once great, it is now reduced considerably allowing new economies to develop and new opportunities to reach people through information and communication technologies to promote health. The rise of ‘Web 2.0’ technologies, itself the subject of a recent special issue of JMIR and a new annual conference[33], reduces the barriers to engagement even further. These tools combine user-created content with easy to operate programs has engaged a new participant (the public) in tobacco control like never before. Social networks like *Facebook* and *MySpace*, or media sharing sites like *YouTube* and *Flickr* are creating new conversations about how to use information technology to help people quit smoking, prevent others from starting, and influencing policy makers on a variety of health issues [34]. It is also creating a new venue for the tobacco industry to attract new customers [35].

The significance of WATI-related research transcends the domain of tobacco cessation, and should be of interest for a wide range of researchers, beyond the tobacco control community. Because of the high prevalence of tobacco abuse (thus large sample sizes), "hard" and comparably easily measurable outcomes (e.g. smoking frequency), and solid research funding for this area, WATI programs have made (and are continuing to make, as showcased by the articles published in this theme issue) important contributions to building the evidence-base for the theory and practice of developing and evaluating Web-based behaviour change programs. The lessons learned in the application of eHealth strategies in the fight against tobacco can be applied in other areas of preventive medicine. More than one third of cancer deaths are attributable to nine modifiable risk factors [36], of which smoking is only one. The other 8 factors are high body mass index, low fruit and vegetable intake, physical inactivity, alcohol use, unsafe sex, urban air pollution, indoor use of solid fuels, and injections from healthcare settings contaminated with hepatitis B or C virus, and at least the first five risk factors are modifiable and can be supported by public eHealth interventions which are very similar to WATI. Interventions addressing these risk factors - in particular those addressing obesity, which is approaching a similar state as tobacco in its threat to population health, have a vast impact on cancer and chronic conditions like diabetes and cardiovascular diseases.

This special issue of the *Journal of Medical Internet Research* presents examples of the state-of-the-art in the research and practice of interventions designed to advance tobacco control through information technology and provides exemplars to guide public health more broadly using eHealth. The collection of papers exploring a range of issues from information searches through to reviewing the state of the literature on WATI or showcasing specific examples. The method of delivery includes traditional websites to mobile phone text messages, while the research designs include qualitative inquiries to randomized controlled trials. All together, the diversity and complexity of how technology can contribute to population health is illustrated, providing a window to how WATI can move us towards a new era of public eHealth and eTobacco control.

## Multimedia Appendix 1

Bibliography WATI Theme Issue (Generic)

## Multimedia Appendix 2

Bibliography WATI Theme Issue (for import into LaTeX and BibDesk Bibliographies)

## Multimedia Appendix 3

Bibliography WATI Theme Issue (for import into Endnote Libraries)

## Multimedia Appendix 4

Bibliography WATI Theme Issue (for import into Endnote, RefMan, Procite and RefWorks Libraries)
