# Supplementary material for: Web-Assisted Tobacco Interventions: Empowering Change in the Global Fight for the Public’s (e)Health
Source: J Med Internet Res. 2008 Nov 25;10(5):e48. doi: 10.2196/jmir.1171 (PMC2630840; doi:10.2196/jmir.1171)
Supplement: Supplementary file 1 [file jmir_v10i5e48_app1.rtf]

An, L. C., B. A. Schillo, et al. (2008). "Utilization of smoking cessation informational, interactive, and online community resources as predictors of abstinence: cohort study." J Med Internet Res 10(5): e55.
	BACKGROUND: The association between greater utilization of Web-assisted tobacco interventions and increased abstinence rates is well recognized. However, there is little information on how utilization of specific website features influences quitting. OBJECTIVE:To determine the association between utilization of informational, interactive, and online community resources (eg. bulletin boards) and abstinence rates, with the broader objective to identify potential strategies for improving outcomes for Web-assisted tobacco interventions. METHODS: In Spring 2004, a cohort of 607 quitplan.com users consented to participate in an evaluation of quitplan.com, a Minnesota branded version of QuitNet.com. We developed utilization measures for different site features: general information, interactive diagnostic tools and quit planning tools, online expert counseling, passive (ie, reading of bulletin boards) and active (ie, public posting) online community engagement, and one-to-one messaging with other virtual community members. Using bivariate, multivariate, and path analyses, we examined the relationship between utilization of specific site features and 30-day abstinence at 6 months. RESULTS: The most commonly used resources were the interactive quit planning tools (used by 77% of site users). Other informational resources (ie, quitting guides) were used more commonly (60% of users) than passive (38%) or active (24%) community features. Online community engagement through one-to-one messaging was low (11%) as was use of online counseling (5%). The 30-day abstinence rate among study participants at 6 months was 9.7% (95% Confidence Interval [CI] 7.3% - 12.1%). In the logistic regression model, neither the demographic data (eg, age, gender, education level, employment, or insurance status) nor the smoking-related data (eg, cigarettes per day, time to first morning cigarette, baseline readiness to quit) nor use of smoking cessation medications entered the model as significant predictors of abstinence. Individuals who used the interactive quit planning tools once, two to three times, or four or more times had an odds of abstinence of 0.65 (95% Confidence Interval [CI] 0.22 - 1.94), 1.87 (95% CI 0.77 - 4.56), and 2.35 (95% CI 1.0 - 5.58), respectively. The use of one-to-one messages (reference = none vs 1 or more) entered the final model as potential predictor for abstinence, though the significance of this measure was marginal (OR = 1.91, 95% CI 0.92 - 3.97, P = .083). In the path analysis, an apparent association between active online community engagement and abstinence was accounted for in large part by increased use of interactive quitting tools and one-to-one messaging. CONCLUSIONS:Use of interactive quitting tools, and perhaps one-to-one messaging with other members of the online community, was associated with increased abstinence rates among quitplan.com users. Designs that facilitate use of these features should be considered.

Balmford, J., R. Borland, et al. (2008). "Patterns of use of an automated interactive personalized coaching program for smoking cessation." J Med Internet Res 10(5): e54.
	BACKGROUND: The QuitCoach, an "expert system" program of tailored advice for smoking cessation developed in Australia, has been publicly available since July 2003, albeit with limited promotion. The program is designed to be used on multiple occasions, guiding the user through the process of smoking cessation in the manner of a "life coach". Email reminders are sent at scheduled intervals to prompt optimal and repeated use. OBJECTIVES: The aims of this study were to characterize QuitCoach users and to determine what characteristics of smokers affect their participation over time. Of particular interest was whether users tend to return following a relapse and, thus, use the program as a tool for relapse prevention or recovery. We also explored patterns of change associated with returns to the site, whether prompted by reminder emails or not prompted at all. METHODS: Between July 2003 and June 2007, 28,247 individuals completed an initial assessment on the QuitCoach, of whom 83.7% (n = 23,656) registered. Data were collected during a 10-minute online questionnaire that all users completed in order to obtain tailored cessation advice. This included questions concerning basic demographic information, quitting history, current smoking status and cigarette consumption, stage of change, and use of pharmacotherapy. RESULTS: The median age of users was 34 years, and 62% were female. Most (96%) were current smokers. Overall, 91% were planning to quit in the next 30 days, and half (49.9%) had set a quit date. Those who had recently relapsed to smoking following a quit attempt made up 37%. Among registered users, 27% returned for a second visit, a median 9 days after their first. Overall, a third visit was completed by 11% and 2% returned within 2 days. Women, older smokers, those who had recently quit, and those using pharmacotherapy were more likely to return. From the second visit on, most people who completed an assessment had quit. Likelihood of responding to a prompt to return was largely unrelated to user characteristics or cessation outcome. CONCLUSIONS: Internet-based programs have considerable potential to reach large numbers of smokers at low cost. The QuitCoach is attracting considerable use, with most using it to make a quit attempt and, for those who continue to use the QuitCoach, to help them stay quit. Nonetheless, most users only visited the site once, suggesting improved strategies are needed for encouraging repeated use.

Bock, B. C., A. L. Graham, et al. (2008). "A review of web-assisted tobacco interventions (WATIs)." J Med Internet Res 10(5): e39.
	BACKGROUND: The Internet has great potential to provide assistance to millions of smokers who seek help with quitting smoking. OBJECTIVE: The goals of this study were to assess the content and the quality of smoking cessation treatments most likely to be encountered by smokers seeking treatment on the Internet and to examine differences in quality between current websites and those reviewed in 2004. METHODS: Internet searches for smoking cessation were designed to mimic the search patterns of most Internet users. PhD-level specialists in tobacco cessation treatments used standardized procedures to review the content of each website, assess the degree to which each site covered key components of evidence-based treatment as described in US national guidelines, determine the accuracy of information presented, and evaluate the use of website interactivity. Results of the current study were compared to results obtained in a prior review. RESULTS: Most websites retrieved in the search met exclusion criteria and were not included in the final analyses in both the current (74%, 65/88) and the prior study (77%, 156/202). In both studies, the majority of websites were excluded because they sold cessation-related products but did not provide treatment recommended by the Public Health Service guidelines. Of the 23 websites included in the current study, 26% (n = 6) provided only minimal coverage (brief mention) of key components of tobacco treatment. However, compared to the earlier study, websites included in the present study scored significantly higher in quality ratings in four areas: providing advice to quit (P = .05), practical counseling (P = .02), and enhancing motivation to quit smoking through personal relevance (P = .05) and risks (P < .001). Most Web-assisted tobacco intervention (WATI) sites (69%, 16/23) contained no inaccurate information. When observed, inaccuracies primarily occurred in content related to pharmacotherapy. The percentage of sites offering at least one interactive feature increased from 39% (18/46) in 2004 to 56% (13/23) in the present study. Despite this improvement, there was a notable underutilization of the interactive capabilities of the Internet to personalize treatment, to connect users with a virtual support system, and to provide follow-up treatment contacts. CONCLUSIONS: While the quality of treatment offered in WATIs has improved since our previous review in 2004, there is substantial room for further improvement to ensure that smokers are offered high-quality, evidence-based treatments. It is not clear what degree of informational detail and interactivity is optimal for Web-based smoking cessation treatments. Additional research is needed to understand how to maximize the interactive capabilities of the Internet to produce and sustain population-based health behavior change.

Brendryen, H., F. Drozd, et al. (2008). "A digital smoking cessation program delivered through internet and cell phone without nicotine replacement (happy ending): randomized controlled trial." J Med Internet Res 10(5): e51.
	BACKGROUND: Happy Ending (HE) is an intense 1-year smoking cessation program delivered via the Internet and cell phone. HE consists of more than 400 contacts by email, Web pages, interactive voice response, and short message service technology. HE includes a craving helpline and a relapse prevention system, providing just-in-time therapy. All the components of the program are fully automated. OBJECTIVE: The objectives were to describe the rationale for the design of HE, to assess the 12-month efficacy of HE in a sample of smokers willing to attempt to quit without the use of nicotine replacement therapy, and to explore the potential effect of HE on coping planning and self-efficacy (prior to quitting) and whether coping planning and self-efficacy mediate treatment effect. METHODS: A two-arm randomized controlled trial was used. Subjects were recruited via Internet advertisements and randomly assigned to condition. Inclusion criteria were willingness to quit on a prescribed day without using nicotine replacement and being aged 18 years or older. The intervention group received HE, and the control group received a 44-page self-help booklet. Abstinence was defined as "not even a puff of smoke, for the last seven days" and was assessed by means of Internet surveys or telephone interviews 1, 3, 6, and 12 months postcessation. The main outcome was repeated point abstinence (ie, abstinence at all four time points). Coping planning and self-efficacy were measured at baseline and at the end of the preparation phase (ie, after 2 weeks of treatment, but prior to cessation day). RESULTS: A total of 290 participants received either the HE intervention (n=144) or the control booklet (n=146). Using intent-to-treat analysis, participants in the intervention group reported clinically and statistically significantly higher repeated point abstinence rates than control participants (20% versus 7%, odds ratio [OR] = 3.43, 95% CI = 1.60-7.34, P = .002). Although no differences were observed at baseline, by the end of the preparation phase, significantly higher levels of coping planning (t(261) = 3.07, P = .002) and precessation self-efficacy (t(261) = 2.63, P = .01) were observed in the intervention group compared with the control group. However, neither coping planning nor self-efficacy mediated long-term treatment effect. For point abstinence 1 month after quitting, however, coping planning and self-efficacy showed a partial mediation of the treatment effect. CONCLUSIONS: This 12-month trial documents a long-term treatment effect of a fully automated smoking cessation intervention without the use of nicotine replacement therapy. The study adds to the promise of using digital media in supporting behavior change.

Cunningham, J. A. (2008). "Access and interest: two important issues in considering the feasibility of web-assisted tobacco interventions." J Med Internet Res 10(5): e37.
	BACKGROUND: Previous research has found that current smokers are less likely to have access to the Internet than nonsmokers. As access to the Internet continues to expand, does this finding remain true? Also, how many smokers are interested in Web-assisted tobacco interventions (WATIs)? These questions are important to determine the potential role that WATIs might play in promoting tobacco cessation. OBJECTIVES: The aims of the study were to determine whether smokers are less likely than nonsmokers to have access to the Internet and to establish the level of interest in WATIs among a representative sample of smokers. METHODS: A random digit dialing telephone survey was conducted of 8467 adult respondents, 18 years and older, in Ontario, Canada from September 2006 to August 2007. All respondents were asked their smoking status and whether they used the Internet (at home or work in the past 12 months; where; how often in the past 12 months). To assess the level of interest in WATIs, current daily smokers were asked whether they would be interested in a confidential program that they could access on the Internet, free of charge, that would allow them to check their smoking and compare it to other Canadians. RESULTS: Smokers were marginally less likely to have used the Internet than nonsmokers (74% vs 81% in the last year), and, of those who had access to the Internet, smokers used the Internet less often than nonsmokers. Overall, 40% of smokers said they would be interested in a WATI. The number of cigarettes smoked per day was unrelated to level of interest in the WATI, but time to first cigarette after waking was. Smokers who used the Internet were more interested in the WATI than smokers who did not use the Internet (46% vs 20%). CONCLUSIONS: While the difference in level of Internet use between smokers and nonsmokers was greatly reduced compared to 2002 and 2004 data, smokers still remain marginally less likely to use the Internet than nonsmokers. Overall, there was a substantial level of interest in the WATI among smokers, in particular among smokers who currently use the Internet. These results indicate that WATIs have a substantial potential audience among smokers, and, given the growing body of evidence regarding their efficacy, there is growing support that WATIs have a significant role to play in promoting tobacco cessation.

Finkelstein, J., O. Lapshin, et al. (2008). "Feasibility of promoting smoking cessation among methadone users using multimedia computer-assisted education." J Med Internet Res 10(5): e33.
	BACKGROUND: The prevalence of smoking is very high among methadone users. As a method of delivering health education, computers can be utilized effectively. However computer-assisted education in methadone users has not been evaluated systematically. OBJECTIVE: This study was aimed at assessing feasibility and patient acceptance of an interactive educational module of a multi-component smoking cessation counseling computer program for former illicit drug users treated in an outpatient methadone clinic. METHODS: The computer-mediated education for hazards of smoking utilized in this study was driven by major constructs of adult learning theories. The program interface was tailored to individuals with minimal computer experience and was implemented on a touch screen tablet PC. The number of consecutive methadone-treated current smokers enrolled in the study was 35. After providing socio-demographic and smoking profiles, the patients were asked to use the educational program for 40 minutes. The impact of the computer-mediated education was assessed by administering a pre- and post-intervention Hazards of Smoking Knowledge Survey (HSKS). An attitudinal survey and semi-structured qualitative interview were used after the educational session to assess the opinions of participants about their educational experience. RESULTS: The computer-mediated education resulted in significant increase of HSKS scores from 60.5 +/- 16.3 to 70.4 +/- 11.7 with t value 3.69 and P < .001. The majority of the patients (78.8%) felt the tablet PC was easy to use, and most of the patients (91.4%) rated the educational experience as good or excellent. After controlling for patient baseline characteristics, the effect of computer-mediated education remained statistically significant. CONCLUSIONS: Computer-assisted education using tablet PCs was feasible, well-accepted, and an effective means of providing hazards of smoking education among methadone users.

Graham, A. L., P. Milner, et al. (2008). "Online advertising as a public health and recruitment tool: comparison of different media campaigns to increase demand for smoking cessation interventions." J Med Internet Res 10(5): e50.
	BACKGROUND: To improve the overall impact (reach x efficacy) of cessation treatments and to reduce the population prevalence of smoking, innovative strategies are needed that increase consumer demand for and use of cessation treatments. Given that 12 million people search for smoking cessation information each year, online advertising may represent a cost-efficient approach to reach and recruit online smokers to treatment. Online ads can be implemented in many forms, and surveys consistently show that consumers are receptive. Few studies have examined the potential of online advertising to recruit smokers to cessation treatments. OBJECTIVE: The aims of the study were to (1) demonstrate the feasibility of online advertising as a strategy to increase consumer demand for cessation treatments, (2) illustrate the tools that can be used to track and evaluate the impact of online advertising on treatment utilization, and (3) highlight some of the methodological challenges and future directions for researchers. METHODS: An observational design was used to examine the impact of online advertising compared to traditional recruitment approaches (billboards, television and radio ads, outdoor advertising, direct mail, and physician detailing) on several dependent variables: (1) number of individuals who enrolled in Web- or telephone-based cessation treatment, (2) the demographic, smoking, and treatment utilization characteristics of smokers recruited to treatment, and (3) the cost to enroll smokers. Several creative approaches to online ads (banner ads, paid search) were tested on national and local websites and search engines. The comparison group was comprised of individuals who registered for Web-based cessation treatment in response to traditional advertising during the same time period. RESULTS: A total of 130,214 individuals responded to advertising during the study period: 23,923 (18.4%) responded to traditional recruitment approaches and 106,291 (81.6%) to online ads. Of those who clicked on an online ad, 9655 (9.1%) registered for cessation treatment: 6.8% (n = 7268) for Web only, 1.1% (n = 1119) for phone only, and 1.2% (n = 1268) for Web and phone. Compared to traditional recruitment approaches, online ads recruited a higher percentage of males, young adults, racial/ethnic minorities, those with a high school education or less, and dependent smokers. Cost-effectiveness analyses compare favorably to traditional recruitment strategies, with costs as low as US $5-$8 per enrolled smoker. CONCLUSIONS: Developing and evaluating new ways to increase consumer demand for evidence-based cessation services is critical to cost-efficiently reduce population smoking prevalence. Results suggest that online advertising is a promising approach to recruit smokers to Web- and telephone-based cessation interventions. The enrollment rate of 9.1% exceeds most studies of traditional recruitment approaches. The powerful targeting capabilities of online advertising present new opportunities to reach subgroups of smokers who may not respond to other forms of advertising. Online advertising also provides unique evaluation opportunities and challenges to determine rigorously its impact and value.

Houston, T. K., J. S. Richman, et al. (2008). "Internet delivered support for tobacco control in dental practice: randomized controlled trial." J Med Internet Res 10(5): e38.
	BACKGROUND: The dental visit is a unique opportunity for tobacco control. Despite evidence of effectiveness in dental settings, brief provider-delivered cessation advice is underutilized. OBJECTIVE: To evaluate an Internet-delivered intervention designed to increase implementation of brief provider advice for tobacco cessation in dental practice settings. METHODS: Dental practices (N = 190) were randomized to the intervention website or wait-list control. Pre-intervention and after 8 months of follow-up, each practice distributed exit cards (brief patient surveys assessing provider performance, completed immediately after the dental visit) to 100 patients. Based on these exit cards, we assessed: whether patients were asked about tobacco use (ASK) and, among tobacco users, whether they were advised to quit tobacco (ADVISE). All intervention practices with follow-up exit card data were analyzed as randomized regardless of whether they participated in the Internet-delivered intervention. RESULTS: Of the 190 practices randomized, 143 (75%) dental practices provided follow-up data. Intervention practices' mean performance improved post-intervention by 4% on ASK (29% baseline, adjusted odds ratio = 1.29 [95% CI 1.17-1.42]), and by 11% on ADVISE (44% baseline, OR = 1.55 [95% CI 1.28-1.87]). Control practices improved by 3% on ASK (Adj. OR 1.18 [95% CI 1.07-1.29]) and did not significantly improve in ADVISE. A significant group-by-time interaction effect indicated that intervention practices improved more over the study period than control practices for ADVISE (P = 0.042) but not for ASK. CONCLUSION: This low-intensity, easily disseminated intervention was successful in improving provider performance on advice to quit. TRIAL REGISTRATION: clinicaltrials.gov NCT00627185, http://www.webcitation.org/5c5Kugvzj.

McKay, H. G., B. G. Danaher, et al. (2008). "Comparing two web-based smoking cessation programs: randomized controlled trial." J Med Internet Res 10(5): e40.
	BACKGROUND: Smoking cessation remains a significant public health problem. Innovative interventions that use the Internet have begun to emerge that offer great promise in reaching large numbers of participants and encouraging widespread behavior change. To date, the relatively few controlled trials of Web-based smoking cessation programs have been limited by short follow-up intervals. OBJECTIVE: We describe the 6-month follow-up results of a randomized controlled trial in which participants recruited online were randomly assigned to either a Web-based smoking cessation program (Quit Smoking Network; QSN) or a Web-based exercise enhancement program (Active Lives) adapted somewhat to encourage smoking cessation. METHODS: The study was a two-arm randomized controlled trial that compared two Web-based smoking cessation programs: (1) the QSN intervention condition presented cognitive-behavioral strategies, and (2) the Active Lives control condition provided participants with guidance in developing a physical activity program to assist them with quitting. The QSN condition provided smoking cessation information and behavior change strategies while the Active Lives condition provided participants with physical activity recommendations and goal setting. The QSN condition was designed to be more engaging (eg, it included multimedia components) and to present much greater content than is typically found in smoking cessation programs. RESULTS: Contrary to our hypotheses, no between-condition differences in smoking abstinence were found at 3- and 6-month follow-up assessments. While participants in the QSN intervention condition spent more time than controls visiting the online program, the median number of 1.0 visit in each condition and the substantial attrition (60.8% at the 6-month follow-up) indicate that participants were not as engaged as we had expected. CONCLUSIONS: Contrary to our hypothesis, our test of two Web-based smoking cessation conditions, an intervention and an attention placebo control, failed to show differences at 3- and 6-month assessments. We explored possible reasons for this finding, including limited engagement of participants and simplifying program content and architecture. Future research needs to address methods to improve participant engagement in online smoking cessation programs. Possible approaches in this regard can include new informed consent procedures that better explain the roles and responsibilities of being a research participant, new program designs that add more vitality (changing content from visit to visit), and new types of reminders pushed out to participants to encourage return visits. Simplifying program content through a combination of enhanced tailoring and information architecture also merits further research attention.

Norman, C. D., S. McIntosh, et al. (2008). "Web-assisted tobacco interventions: empowering change in the global fight for the public's (e)Health." J Med Internet Res 10(5): e48.
	Tobacco control in the 21(st) century faces many of the same challenges as in the past, but in different contexts, settings and enabled by powerful new tools including those delivered by information and communication technologies via computer, videocasts, and mobile handsets to the world. Building on the power of electronic networks, Web-assisted tobacco interventions (WATI) provide a vehicle for delivering tobacco prevention, cessation, social support and training opportunities on-demand and direct to practitioners and the public alike. The Framework Convention on Tobacco Control, the world's first global public health treaty, requires that all nations develop comprehensive tobacco control strategies that include provision of health promotion information, population interventions, and decision-support services. WATI research and development has evolved to provide examples of how eHealth can address all of these needs and provide exemplars for other areas of public health to follow. This paper discusses the role of WATI in supporting tobacco control and introduces a special issue of the Journal of Medical Internet Research that broadens the evidence base and provides illustrations of how new technologies can support health promotion and population health overall, empowering change and ushering in a new era of public eHealth.

Rabius, V., K. J. Pike, et al. (2008). "Comparing internet assistance for smoking cessation: 13-month follow-up of a six-arm randomized controlled trial." J Med Internet Res 10(5): e45.
	BACKGROUND: Although many smokers seek Internet-based cessation assistance, few studies have experimentally evaluated long-term cessation rates among cigarette smokers who receive Internet assistance in quitting. OBJECTIVE: The purpose of this study is to describe long-term smoking cessation rates associated with 6 different Internet-based cessation services and the variation among them, to test the hypothesis that interactive and tailored Internet services yield higher long-term quit rates than more static Web-posted assistance, and to explore the possible effects of level of site utilization and a self-reported indicator of depression on long-term cessation rates. METHOD: In 2004-05, a link was placed on the American Cancer Society (ACS) website for smokers who wanted help in quitting via the Internet. The link led smokers to the QuitLink study website, where they could answer eligibility questions, provide informed consent, and complete the baseline survey. Enrolled participants were randomly assigned to receive emailed access to one of five tailored interactive sites provided by cooperating research partners or to a targeted, minimally interactive ACS site with text, photographs, and graphics providing stage-based quitting advice and peer modeling. RESULTS: 6451 of the visitors met eligibility requirements and completed consent procedures and the baseline survey. All of these smokers were randomly assigned to one of the six experimental groups. Follow-up surveys done online and via telephone interviews at approximately 13 months after randomization yielded 2468 respondents (38%) and found no significant overall quit rate differences among those assigned to the different websites (P = .15). At baseline, 1961 participants (30%) reported an indicator of depression. Post hoc analyses found that this group had significantly lower 13-month quit rates than those who did not report the indicator (all enrolled, 8% vs 12%, P < .001; followed only, 25% vs 31%, P = .003). When the 4490 participants (70%) who did not report an indicator of depression at baseline were separated for analysis, the more interactive, tailored sites, as a whole, were associated with higher quitting rates than the less interactive ACS site: 13% vs 10% (P = .04) among 4490 enrolled and 32% vs 26% (P = .06) among 1798 followed. CONCLUSIONS: These findings show that Internet assistance is attractive and potentially cost-effective and suggest that tailored, interactive websites may help cigarette smokers who do not report an indicator of depression at baseline to quit and maintain cessation.

Stoddard, J. L., E. M. Augustson, et al. (2008). "Effect of adding a virtual community (bulletin board) to smokefree.gov: randomized controlled trial." J Med Internet Res 10(5): e53.
	BACKGROUND: Demand for online information and help exceeds most other forms of self-help. Web-assisted tobacco interventions (WATIs) offer a potentially low-cost way to reach millions of smokers who wish to quit smoking and to test various forms of online assistance for use/utilization and user satisfaction. OBJECTIVES: Our primary aim was to determine the utilization of and satisfaction with 2 versions of a smoking cessation website (smokefree.gov), one of which included an asynchronous bulletin board (BB condition). A secondary goal was to measure changes in smoking behavior 3 months after enrollment in the study. METHODS: All participants were adult federal employees or contractors to the federal government who responded to an email and indicated a willingness to quit smoking in 30 days. We randomly assigned participants to either the BB condition or the publicly available version--usual care (UC)--and then assessed the number of minutes of website use and satisfaction with each condition as well as changes in smoking behavior. RESULTS: Among the 1375 participants, 684 were randomized to the BB intervention, and 691 to the control UC condition. A total of 39.7% returned a follow-up questionnaire after 3 months, with similar rates across the two groups (UC: n=279, 40.3%; BB: n=267, 39.0%). Among those respondents assigned to the BB condition, only 81 participants (11.8%) elected to view the bulletin board or post a message, limiting our ability to analyze the impact of bulletin board use on cessation. Satisfaction with the website was high and did not differ significantly between conditions (UC: 90.2%, BB: 84.9%, P= .08). Utilization, or minutes spent on the website, was significantly longer for the BB than the UC condition (18.0 vs 11.1, P = .01) and was nearly double for those who remained in the study (21.2) than for those lost to follow-up (9.6, P< .001). Similar differences were observed between those who made a serious quit attempt versus those who did not (22.4 vs 10.4, P= .02) and between those with a quit date on or a few days prior to the enrollment date versus those with a later quit date (29.4 vs 12.5, P = .001). There were no statistically significant differences in quit rates between the BB and UC group, both in intent-to-treat analysis (ITT) and in analyzing the adherence subgroup (respondents) only. Combined across the UC and BB groups, 7-day abstinence was 6.8% with ITT and 17.6% using only participants in the follow-up (adherence). For participants who attempted to quit within a few days of study entry (vs 30 days), quit rates were 29.6% (ITT) and 44.4% (adherence). CONCLUSIONS: Quit rates for participants were similar to other WATIs, with the most favorable outcomes demonstrated by smokers ready to quit at the time of enrolling in the trial and smokers using pharmacotherapy. Utilization of the asynchronous bulletin board was lower than expected, and did not have an impact on outcomes (quit rates). Given the demand for credible online resources for smoking cessation, future studies should continue to evaluate use of and satisfaction with Web features and to clarify results in terms of time since last cigarette as well as use of pharmacotherapy.

Strecher, V. J., J. McClure, et al. (2008). "The role of engagement in a tailored web-based smoking cessation program: randomized controlled trial." J Med Internet Res 10(5): e36.
	BACKGROUND: Web-based programs for health promotion, disease prevention, and disease management often experience high rates of attrition. There are 3 questions which are particularly relevant to this issue. First, does engagement with program content predict long-term outcomes? Second, which users are most likely to drop out or disengage from the program? Third, do particular intervention strategies enhance engagement? OBJECTIVE: To determine: (1) whether engagement (defined by the number of Web sections opened) in a Web-based smoking cessation intervention predicts 6-month abstinence, (2) whether particular sociodemographic and psychographic groups are more likely to have lower engagement, and (3) whether particular components of a Web-based smoking cessation program influence engagement. METHODS: A randomized trial of 1866 smokers was used to examine the efficacy of 5 different treatment components of a Web-based smoking cessation intervention. The components were: high- versus low-personalized message source, high- versus low-tailored outcome expectation, efficacy expectation, and success story messages. Moreover, the timing of exposure to these sections was manipulated, with participants randomized to either a single unified Web program with all sections available at once, or sequential exposure to each section over a 5-week period of time. Participants from 2 large health plans enrolled to receive the online behavioral smoking cessation program and a free course of nicotine replacement therapy (patch). The program included: an introduction section, a section focusing on outcome expectations, 2 sections focusing on efficacy expectations, and a section with a narrative success story (5 sections altogether, each with multiple screens). Most of the analyses were conducted with a stratification of the 2 exposure types. Measures included: sociodemographic and psychosocial characteristics, Web sections opened, perceived message relevance, and smoking cessation 6-months following quit date. RESULTS: The total number of Web sections opened was related to subsequent smoking cessation. Participants who were younger, were male, or had less formal education were more likely to disengage from the Web-based cessation program, particularly when the program sections were delivered sequentially over time. More personalized source and high-depth tailored self-efficacy components were related to a greater number of Web sections opened. A path analysis model suggested that the impact of high-depth message tailoring on engagement in the sequentially delivered Web program was mediated by perceived message relevance. CONCLUSIONS: Results of this study suggest that one of the mechanisms underlying the impact of Web-based smoking cessation interventions is engagement with the program. The source of the message, the degree of message tailoring, and the timing of exposure appear to influence Web-based program engagement.

Whittaker, R., R. Maddison, et al. (2008). "A multimedia mobile phone-based youth smoking cessation intervention: findings from content development and piloting studies." J Med Internet Res 10(5): e49.
	BACKGROUND: While most young people who smoke want to quit, few access cessation support services. Mobile phone-based cessation programs are ideal for young people: mobile phones are the most common means of peer communication, and messages can be delivered in an anonymous manner, anywhere, anytime. Following the success of our text messaging smoking cessation program, we developed an innovative multimedia mobile phone smoking cessation intervention. OBJECTIVE: The aim of the study was to develop and pilot test a youth-oriented multimedia smoking cessation intervention delivered solely by mobile phone. METHODS: Development included creating content and building the technology platform. Content development was overseen by an expert group who advised on youth development principles, observational learning (from social cognitive theory), effective smoking cessation interventions, and social marketing. Young people participated in three content development phases (consultation via focus groups and an online survey, content pre-testing, and selection of role models). Video and text messages were then developed, incorporating the findings from this research. Information technology systems were established to support the delivery of the multimedia messages by mobile phone. A pilot study using an abbreviated 4-week program of video and text content tested the reliability of the systems and the acceptability of the intervention. RESULTS: Approximately 180 young people participated in the consultation phase. There was a high priority placed on music for relaxation (75%) and an interest in interacting with others in the program (40% would read messages, 36% would read a blog). Findings from the pre-testing phase (n = 41) included the importance of selecting "real" and "honest" role models with believable stories, and an interest in animations (37%). Of the 15 participants who took part in the pilot study, 13 (87%) were available for follow-up interviews at 4 weeks: 12 participants liked the program or liked it most of the time and found the role model to be believable; 7 liked the role model video messages (5 were unsure); 8 used the extra assistance for cravings; and 9 were happy with two messages per day. Nine participants (60%) stopped smoking during the program. Some technical challenges were encountered during the pilot study. CONCLUSIONS: A multimedia mobile phone smoking cessation program is technically feasible, and the content developed is appropriate for this medium and is acceptable to our target population. These results have informed the design of a 6-month intervention currently being evaluated for its effectiveness in increasing smoking cessation rates in young people.

Zbikowski, S. M., J. Hapgood, et al. (2008). "Phone and web-based tobacco cessation treatment: real-world utilization patterns and outcomes for 11,000 tobacco users." J Med Internet Res 10(5): e41.
	BACKGROUND: Phone-based tobacco cessation programs have been proven effective and widely adopted. Web-based solutions exist; however, the evidence base is not yet well established. Many cessation treatments are commercially available, but few integrate the phone and Web for delivery and no published studies exist for integrated programs. OBJECTIVE: This paper describes a comprehensive integrated phone/Web tobacco cessation program and the characteristics, experience, and outcomes of smokers enrolled in this program from a real-world evaluation. METHODS: We tracked program utilization (calls completed, Web log-ins), quit status, satisfaction, and demographics of 11,143 participants who enrolled in the Free & Clear Quit For Life Program between May 2006 and October 2007. All participants received up to five proactive phone counseling sessions with Quit Coaches, unlimited access to an interactive website, up to 20 tailored emails, printed Quit Guides, and cessation medication information. The program was designed to encourage use of all program components rather than asking participants to choose which components they wanted to use while quitting. RESULTS: We found that participants tended to use phone services more than Web services. On average, participants completed 2-2.5 counseling calls and logged in to the online program 1-2 times. Women were more adherent to the overall program; women utilized Web and phone services significantly (P = .003) more than men. Older smokers (> 26 years) and moderate smokers (15-20 cigarettes/day) utilized services more (P < .001) than younger (< 26 years) and light or heavy smokers. Satisfaction with services was high (92% to 95%) and varied somewhat with Web utilization. Thirty-day quit rates at the 6-month follow-up were 41% using responder analysis and 21% using intent-to-treat analysis. Web utilization was significantly associated with increased call completion and tobacco abstinence rates at the 6-month follow-up evaluation. CONCLUSIONS: This paper expands our understanding of a real-world treatment program combining two mediums, phone and Web. Greater adherence to the program, as defined by using both the phone and Web components, is associated with higher quit rates. This study has implications for reaching and treating tobacco users with an integrated phone/Web program and offers evidence regarding the effectiveness of integrated cessation programs.
